# Determination of Lifestyle Habits Correlating to the Prevalence of Hypertension, Diabetes, and Dyslipidemia by the Analysis of Health-Related Questionnaire Datasets in Japanese Nationwide Open Data

**DOI:** 10.7759/cureus.77105

**Published:** 2025-01-07

**Authors:** Yukinori Nagakura, Fumiko Yamaki, Hiroshi Saimaru, Yoshio Kase

**Affiliations:** 1 Research Center for Pharmaceutical Carrier Education, Faculty of Pharmacy, Musashino University, Nishitokyo, JPN

**Keywords:** analysis of open data, diabetes, dyslipidemia, hypertension, lifestyle habits

## Abstract

Introduction: The National Database of Health Insurance Claims and Specific Health Checkups (NDB) Open Data Japan provides a nationwide health-related dataset based on region. This study aimed to identify lifestyle habits that correlated with the prevalence of hypertension, diabetes, and dyslipidemia by analyzing a dataset.

Methods: Data from 28.9 million respondents regarding lifestyle habits were collected in the fiscal year 2020 and provided in the 8^th^ NDB Open Data Japan. Medication status for hypertension, diabetes, and dyslipidemia was used to determine the prevalence of each disorder. Responses to lifestyle habit questions were used as lifestyle variables. Pearson's correlation coefficient (r) was calculated to determine the relationships between variables.

Results: Lifestyle habits that had a moderate or larger correlation with the prevalence of each disorder were identified by setting the criterion |r| > 0.5. Smoking, weight gain, chewing condition, eating speed, snacking, and alcohol consumption were associated with the prevalence of hypertension. Smoking, weight gain, and chewing conditions correlated with the prevalence of diabetes. No single lifestyle habit showed correlations above the set criterion for dyslipidemia prevalence.

Conclusion: Due to the diversity of lifestyle habits of residents within each of the 47 Japanese prefectures, the prefecture-based dataset in NDB Open Data Japan is pragmatic and useful for epidemiologically investigating the association between lifestyle habits and the prevalence of disorders of interest. It would be important to raise the alarm about the lifestyle habits identified in the present study to reduce the risk of developing the corresponding disorders.

## Introduction

Hypertension, diabetes, and dyslipidemia are representative disorders whose onset and progression are closely associated with lifestyle habits [1-3]. As they are major risk factors for a series of serious cardiovascular diseases [4,5], preventing their onset and progression by appropriately controlling lifestyle habits is crucial for a long and healthy life. Although it has been suggested that various lifestyle habits are associated with the development of these disorders, no definitive conclusion has been reached regarding their levels of involvement. Furthermore, few studies have investigated lifestyle habits related to these three disorders in a single study.

Japan's territory stretches widely from north to south and east to west and is divided into 47 administrative units termed prefectures [6]. Each prefecture has a diverse topography, climate, unique commerce, culture, and individual way of life. Japanese government ministries and agencies annually collect and compile prefecture-specific data in a variety of categories throughout the country, which are published online as open data [7]. Specifically, the National Database of Health Insurance Claims and Specific Health Checkups (NDB) Open Data Japan provides health-associated real-world datasets useful for epidemiological studies [8]. Lifestyle habit-associated questionnaires are routinely asked as part of health check-ups of recipients aged 40 to 74 years. The 8^th^ NDB Open Data Japan contains data from 28.9 million responses to these questionnaires in the fiscal year 2020 [9]. As lifestyle habits vary significantly by prefecture [10], the analysis of datasets aggregated by prefecture in the NDB Open Data Japan is an efficient research approach for identifying lifestyle habits associated with health outcomes of interest. One study revealed by analyzing the datasets that the prevalence of chronic constipation is closely associated with the use of antihypertensive drugs [11]. We also recently identified several lifestyle habits that are closely related to the prevalence of diseases of interest (i.e., benign prostatic hyperplasia [12] and chronic pain [13]) by analyzing the datasets.

By analyzing the answers to lifestyle habit-associated questionnaires in the 8^th^ NDB Open Data Japan, the present study aimed to identify lifestyle habits that are associated with the prevalence of hypertension, diabetes, and dyslipidemia and contribute to maintaining healthy lifestyles. While previous studies have investigated lifestyle habits associated with individual diseases, few have comprehensively examined correlations across hypertension, diabetes, and dyslipidemia in a single study using a unified dataset. This study addresses this gap by utilizing a comprehensive and nationwide dataset.

## Materials and methods

This cross-sectional epidemiological study investigated the correlation between lifestyle variables and the prevalence of hypertension, diabetes, and dyslipidemia.

Data source and variables

This study analyzed the NDB Open Data Japan, a database built and managed by the Ministry of Health, Labor, and Welfare, which grasps medical and health trends at the national level. Because Japan has implemented a universal health insurance system, all individuals aged 40-74 years are provided with the opportunity to undergo a specific health checkup operated by public health insurers every fiscal year. As part of the checkup, questionnaires regarding lifestyle habits are routinely administered. The results of the check-up, including answers to the questionnaires, are published online in NDB Open Data Japan as open data that removes any personally identifiable information. This study analyzed data from 28.9 million respondents to questionnaires regarding lifestyle habits in the 8^th^ NDB Open Data Japan, which were collected in the fiscal year 2020 and aggregated by prefecture [9]. Thus, the analysis was conducted using the prefecture as the unit. Medication status for hypertension, diabetes, and dyslipidemia (i.e., the prefecture-level average percentage of health check-up recipients who answered that they were taking medication) was used as a variable for the prevalence of each disorder. Meanwhile, the answers to lifestyle habit-associated questionnaires (i.e., the prefecture-level average percentage of health checkup recipients who selected each answer option) were utilized as variables for lifestyle habits. Because the age composition of the residents varies from prefecture to prefecture, the raw data of answers to questionnaires (i.e., average percentage in the prefecture) were standardized for age using the formula: Standardized data = (∑ age-specific raw data in a five-year age group × standard population in that age group)/(total population in standard population), where the Japanese demographic composition [14] was used as the standard population.

Statistical analysis

The correlations between the variables for the prevalence of each lifestyle-related disorder and lifestyle habits were calculated. Pearson’s correlation coefficient (r) for all possible pairs of variables was determined. According to a guideline for interpreting the strength of r, 0 ≤ |r| ≤ 0.3 was interpreted as negligible correlation, 0.3 < |r| ≤ 0.5 as low correlation, 0.5 < |r| ≤ 0.7 as medium correlation, 0.7 < |r| ≤ 0.9 as high correlation, and 0.9 < |r| ≤ 1 as very high correlation [15]. The statistical significance of r was tested using a t-test, and p-values less than 0.05 were regarded as significant. Lifestyle habits that had more than a medium correlation (i.e., 0.5 < |r|) to the prevalence of each lifestyle-related disorder with statistical significance (i.e., p < 0.05) were focused on, although those that had negligible or weak correlation (i.e., |r| ≤ 0.5) were not screened. Statistical analysis was conducted using the Bell Curve for Excel version 3.20 (Social Survey Research Information Co., Ltd., Tokyo, Japan), and p-values less than 0.05 were regarded as statistically significant.

## Results

The questionnaires regarding lifestyle habits, answer options for each questionnaire, and nationwide average percentages of health checkup recipients who selected each answer are summarized in Table 1.

**Table 1:** Variables for analysis (): Answer options in each question; Nationwide %: nationwide average percentages of respondents (adults aged between 40–74 years) selecting each answer, which was calculated using the datasets in 8^th^ NDB Open Data Japan [9].

Category		Question (answer options)	Answer selected	Nationwide %	
				Males	Females
Drug medication status		Are you currently taking medication to reduce blood pressure? (Yes/No)	Yes	24.2	17.4
		Are you currently taking insulin injections or other medications to reduce your blood glucose level? (Yes/No)	Yes	7.6	3.5
		Are you currently taking medication to reduce cholesterol or triglycerides? (Yes/No)	Yes	14.0	15.7
Lifestyle habits	Smoking	Do you habitually smoke cigarettes (“habitual smoker” smokes 100 or more cigarettes in total or for six months or more, and has smoked in the last month)? (Yes/No)	Yes	32.5	9.7
	Weight gain	Have you gained 10 kg or more since you were 20 years old? (Yes/No)	Yes	48.5	29.2
	Exercise	Have you been habitually doing slightly sweaty exercise for 30 minutes or more at least twice a week for over a year? (Yes/No)	Yes	29.3	23.8
	Walking	Have you been walking or engaging in any equivalent physical activity for over an hour per day in everyday life? (Yes/No)	Yes	41.1	42.5
	Walking speed	Do you walk faster than those of the same age and gender? (Yes/No)	Yes	47.8	44.7
	Chewing condition	Which of the following describes your condition when you chew your meal? (A1) Chew anything (A2) Sometimes hard to chew (A3) Hardly chew	A1	81.1	84.5
			A2	17.9	15.0
			A3	1.0	0.4
	Eating speed	Is your eating speed faster than others? (A1) Faster (A2) Similar (A3) Slower	A1	36.1	27.3
			A2	57.4	64.2
			A3	6.5	8.6
	Late dinner	Do you eat dinner within two hours before going to bed three times or more per week? (Yes/No)	Yes	34.2	18.2
	Snacking	Do you have any snacks or sweet drinks other than the three meals? (A1) Every day (A2) Sometimes (A3) Rarely or never	A1	13.2	29.4
			A2	55.7	56.8
			A3	31.3	13.8
	Breakfast skipping	Do you skip breakfast three or more times per week? (Yes/No)	Yes	21.7	12.9
	Alcohol drinking frequency	How often do you drink alcohol (e.g., sake, shochu, beer, whisky, wine)? (A1) Every day (A2) Sometimes (A3) Rarely or never	A1	37.7	14.3
			A2	30.1	27.5
			A3	32.2	58.2
	Alcohol drinking amount	How much alcohol do you drink on a given day in terms of sake? (180 mL of sake is equivalent to 500 mL of beer, 80 mL of shochu, 60 mL of whisky, 240 mL of wine) (A1) < 180 mL (A2) 180–360 mL (A3) 360–540 mL (A4) 540 mL <	A1	42.9	75.1
			A2	34.5	18.8
			A3	17.0	4.8
			A4	5.6	1.2

Correlations between the variables for the prevalence of hypertension and lifestyle habits are shown in Table 2.

**Table 2:** The correlation between antihypertensive medication and lifestyle habits Bold face represents |*r*| > 0.5 by Pearson’s correlation analysis.

Questions	Answer selected	Males		Females	
		*r *(95% confidence interval)	P	*r* (95% confidence interval)	P
Antidiabetic medication	Yes	0.7231 (0.5502 - 0.8366)	<0.0001	0.7812 (0.6370 - 0.8726)	<0.0001
Antidyslipidemic medication	Yes	0.1796 (-0.1134 - 0.4439)	0.2270	0.2996 (0.0136 - 0.5403)	0.0408
Smoking	Yes	0.5676 (0.3350 - 0.7350)	<0.0001	0.2659 (-0.0231 - 0.5138)	0.0709
Weight gain	Yes	0.2765 (-0.0116 - 0.5222)	0.0599	0.6053 (0.3851 - 0.7603)	<0.0001
Exercise	Yes	0.0019 (-0.2854 - 0.2889)	0.9900	-0.2848 (-0.5287 - 0.0026)	0.0523
Walking	Yes	0.1240 (-0.1692 - 0.3970)	0.4065	-0.1338 (-0.4054 - 0.1595)	0.3698
Walking speed	Yes	-0.1236 (-0.3967 - 0.1696)	0.4077	-0.1971 (-0.4584 - 0.0954)	0.1841
Chewing condition	Chew anything	-0.5113 (-0.6962 - -0.2627)	0.0002	-0.4954 (-0.6851 - -0.2427)	0.0004
	Sometimes hard to chew	0.5296 (0.2859 - 0.7090)	0.0001	0.5026 (0.2517 - 0.6901)	0.0003
	Hardly chew	0.0821 (-0.2100 - 0.3608)	0.5832	0.1110 (-0.1820 - 0.3858)	0.4578
Eating speed	Faster	-0.4325 (-0.6401 - -0.1659)	0.0024	-0.0149 (-0.3008 - 0.2734)	0.9208
	Similar	0.5570 (0.3213 - 0.7278)	<0.0001	0.1522 (-0.1412 - 0.4209)	0.3072
	Slower	-0.5224 (-0.7040 - -0.2768)	0.0002	-0.4712 (-0.6679 - -0.2128)	0.0008
Late dinner	Yes	0.0778 (-0.2141 - 0.3570)	0.6031	0.2686 (-0.0201 - 0.5160)	0.0679
Snacking	Every day	-0.5949 (-0.7534 - -0.3711)	<0.0001	-0.4372 (-0.6436 - -0.1716)	0.0021
	Sometimes	-0.0466 (-0.3294 - 0.2438)	0.7556	0.3006 (0.0147 - 0.5411)	0.0400
	Rarely or never	0.4327 (0.1662 - 0.6403)	0.0024	0.3177 (0.0336 - 0.5543)	0.0295
Breakfast skipping	Yes	0.3247 (0.0414 - 0.5597)	0.0260	0.2675 (-0.0213 - 0.5151)	0.0691
Alcohol drinking frequency	Every day	0.3317 (0.0492 - 0.5650)	0.0228	-0.2859 (-0.5296 - 0.0013)	0.0514
	Sometimes	0.3415 (0.0602 - 0.5725)	0.0188	0.1520 (-0.1413 - 0.4208)	0.3077
	Rarely or never	-0.6447 (-0.7863 - -0.4388)	<0.0001	0.0497 (-0.2409 - 0.3321)	0.7403
Alcohol drinking amount	< 180 mL	-0.6068 (-0.7613 - -0.3871)	<0.0001	-0.2872 (-0.5306 - -0.0001)	0.0503
	180–360 mL	0.5866 (0.3601 - 0.7478)	<0.0001	0.3294 (0.0467 - 0.5633)	0.0237
	360–540 mL	0.4213 (0.1526 - 0.6320)	0.0032	0.1392 (-0.1542 - 0.4099)	0.3509
	540 mL <	0.2665 (-0.0224 - 0.5143)	0.0702	0.1920 (-0.1007 - 0.4541)	0.1961

Lifestyle habits, which were positively correlated with the prevalence of hypertension above the criterion |r| > 0.5 by Pearson’s correlation analysis (marked in bold face), were antidiabetic medications (both genders), smoking (males), body weight gain (females), sometimes hard to chew (both genders), similar eating speed (males), and 180-360 mL of alcohol drinking (males). On the other hand, lifestyle habits, which were negatively correlated with the prevalence of hypertension, were chewing anything (males), slower eating speed (males), snacking every day (males), rarely or never drinking alcohol (males), and < 180 mL of alcohol drinking (males). The correlations between the prevalence of diabetes and lifestyle habits are shown in Table 3.

**Table 3:** The correlation between antidiabetic medication and lifestyle habits Bold face represents |*r*| > 0.5 by Pearson’s correlation analysis.

Questions	Answer selected	Males		Females	
		*r* (95% confidence interval)	P	*r *(95% confidence interval)	P
Antihypertensive medication	Yes	0.7231 (0.5502 - 0.8366)	<0.0001	0.7812 (0.6370 - 0.8726)	<0.0001
Antidyslipidemic medication	Yes	0.1796 (-0.1134 - 0.4439)	0.2270	0.2996 (0.0136- 0.5403)	0.0408
Smoking	Yes	0.6343 (0.4245- 0.7795)	<0.0001	0.2659 (-0.1453 - 0.4175)	0.3206
Weight gain	Yes	0.3216 (0.0379 - 0.5573)	0.0275	0.6539 (0.4516 - 0.7923)	<0.0001
Exercise	Yes	-0.0597 (-0.3410 - 0.2314)	0.6901	-0.1842 (-0.4477 - 0.1087)	0.2152
Walking	Yes	0.0703 (-0.2213 - 0.3504)	0.6385	-0.0305 (-0.3149 - 0.2589)	0.8385
Walking speed	Yes	-0.2069 (-0.4664 - 0.0853)	0.1628	-0.2122 (-0.4707 - 0.0798)	0.1521
Chewing condition	Chew anything	-0.5733 (-0.7388 - -0.3425)	<0.0001	-0.5124 (-0.6970 - -0.2641)	0.0002
	Sometimes hard to chew	0.5707 (0.3391 - 0.7371)	<0.0001	0.5080 (0.2586 - 0.6939)	0.0003
	Hardly chew	0.2150 (-0.0769 - 0.4730)	0.1467	0.2158 (-0.0761 - 0.4736)	0.1451
Eating speed	Faster	-0.2150 (-0.4730 - 0.0769)	0.1466	0.1358 (-0.1575 - 0.4071)	0.3627
	Similar	0.3427 (0.0616 - 0.5734)	0.0184	-0.0082 (-0.2947 - 0.2796)	0.9564
	Slower	-0.4982 (-0.6870 - -0.2462)	0.0004	-0.4021 (-0.6179 - -0.1300)	0.0051
Late dinner	Yes	0.0767 (-0.2152 - 0.3560)	0.6085	0.2959 (0.0096 - 0.5374)	0.0434
Snacking	Every day	-0.4462 (-0.6501 - -0.1824)	0.0017	-0.4045 (0.6197 - -0.1327)	0.0048
	Sometimes	-0.0092 (-0.2956 - 0.2787)	0.9510	0.3419 (0.0606 - 0.5728)	0.0187
	Rarely or never	0.3054 (0.0199 - 0.5448)	0.0369	0.1961 (-0.0965 - 0.4575)	0.1864
Breakfast skipping	Yes	0.3754 (0.0955 - 0.6003)	0.0102	0.3193 (0.0354 - 0.5555)	0.0287
Alcohol drinking frequency	Every day	0.1511 (-0.1422 - 0.4201)	0.3106	-0.3980 (-0.6149 - -0.1252)	0.0056
	Sometimes	0.2364 (-0.0545 - 0.4903)	0.1097	-0.0358 (-0.3197 - 0.2539)	0.8109
	Rarely or never	-0.3654 (-0.5906 - -0.0875)	0.0115	0.2291 (-0.0622 - 0.4844)	0.1214
Alcohol drinking amount	< 180 mL	-0.4597 (-0.6598 - -0.1988)	0.0012	-0.2790 (-0.5242 - 0.0089)	0.0575
	180–360 mL	0.4411 (0.1763 - 0.6464)	0.0019	0.3033 (0.0177 - 0.5432)	0.0382
	360–540 mL	0.2907 (0.0038 - 0.5333)	0.0874	0.1698 (-0.1234 - 0.4357)	0.3509
	540 mL <	0.2521 (-0.0378 - 0.5029)	0.0474	0.2093 (-0.0829 - 0.4683)	0.1961

Lifestyle habits that were positively correlated with the prevalence of diabetes above the criterion were antihypertensive medication (both genders), smoking (males), body weight gain (females), and sometimes hard to chew (both genders), whereas chewing anything (males) was negatively correlated with the prevalence of diabetes. The correlations between the prevalence of dyslipidemia and lifestyle habits are presented in Table 4.

**Table 4:** The correlation between antidyslipidemic medication and lifestyle habits

Questions	Answer selected	Males		Females	
		*r* (95% confidence interval)	P	*r* (95% confidence interval)	P
Antihypertensive medication	Yes	0.1796 (-0.1134 - 0.4439)	0.2270	0.2996 (0.0136 - 0.5403)	0.0408
Antidiabetic medication	Yes	0.1072 (-0.1857 - 0.3826)	0.4732	0.0533 (-0.2375 - 0.3353)	0.7221
Smoking	Yes	-0.0750 (-0.3546 - 0.2168)	0.6161	0.0917 (-0.2007 - 0.3692)	0.5397
Weight gain	Yes	0.1637 (-0.1295 - 0.4307)	0.2714	-0.1312 (-0.4032 - 0.1621)	0.3794
Exercise	Yes	0.0452 (-0.2452 - 0.3281)	0.7631	-0.3637 (-0.5893 - -0.0855)	0.0120
Walking	Yes	-0.0598 (-0.3411 - 0.2314)	0.6898	-0.3186 (-0.5550 - -0.0346)	0.0291
Walking speed	Yes	0.0983 (-0.1944 - 0.3749)	0.5110	-0.2713 (-0.5181 - 0.0172)	0.0651
Chewing condition	Chew anything	0.0933 (-0.1992 - 0.3706)	0.5327	-0.0611 (-0.3423 - 0.2301)	0.6832
	Sometimes hard to chew	-0.0586 (-0.3400 - 0.2325)	0.6956	0.0870 (-0.2053 - 0.3651)	0.5608
	Hardly chew	-0.2173 (-0.4749 - 0.0745)	0.1423	-0.2024 (-0.4627 - 0.0900)	0.1724
Eating speed	Faster	-0.0893 (-0.3671 - 0.2031)	0.5505	-0.2757 (-0.5216 - 0.0124)	0.0607
	Similar	0.1156 (-0.1774 - 0.3899)	0.4389	0.3438 (0.0629 - 0.5743)	0.0180
	Slower	-0.1101 (-0.3851 - 0.1829)	0.4613	-0.2981 (-0.5392 - -0.0120)	0.0418
Late dinner	Yes	0.0001 (-0.2870 - 0.2873)	0.9992	-0.3522 (-0.5807 - -0.0724)	0.0152
Snacking	Every day	-0.0512 (-0.3334 - 0.2395)	0.7326	-0.1007 (-0.3770 - 0.1920)	0.5004
	Sometimes	0.0469 (-0.2435 - 0.3296)	0.7542	-0.0542 (-0.3361 - 0.2367)	0.7177
	Rarely or never	-0.0008 (-0.2879 - 0.2865)	0.9959	0.2625 (-0.0266 - 0.5112)	0.0746
Breakfast skipping	Yes	0.0880 (-0.2043 - 0.3659)	0.5563	-0.2070 (-0.4664 - 0.0852)	0.1627
Alcohol drinking frequency	Every day	-0.3180 (-0.5545 - -0.0340)	0.0294	-0.2213 (-0.4781 - 0.0704)	0.1350
	Sometimes	0.4486 (0.1853 - 0.6518)	0.0016	0.1069 (-0.1860 - 0.3823)	0.4747
	Rarely or never	-0.0705 (-0.3506 - 0.2211)	0.6377	-0.0705 (-0.2450 - 0.3283)	0.7619
Alcohol drinking amount	< 180 mL	0.0479 (-0.2426 - 0.3305)	0.7494	0.1457 (-0.1476 - 0.4155)	0.3283
	180–360 mL	-0.0281 (-0.3127 - 0.2612)	0.8514	-0.0679 (-0.3483 - 0.2236)	0.6503
	360–540 mL	-0.0833 (-0.3618 - 0.2089)	0.8963	-0.2970 (-0.5383 - -0.0108)	0.0426
	540 mL <	0.0195 (-0.2691 - 0.3050)	0.5778	-0.2213 (-0.4781 - 0.0703)	0.1350

No lifestyle habit variables met the criterion |*r*| > 0.5.

**Figure 1:**
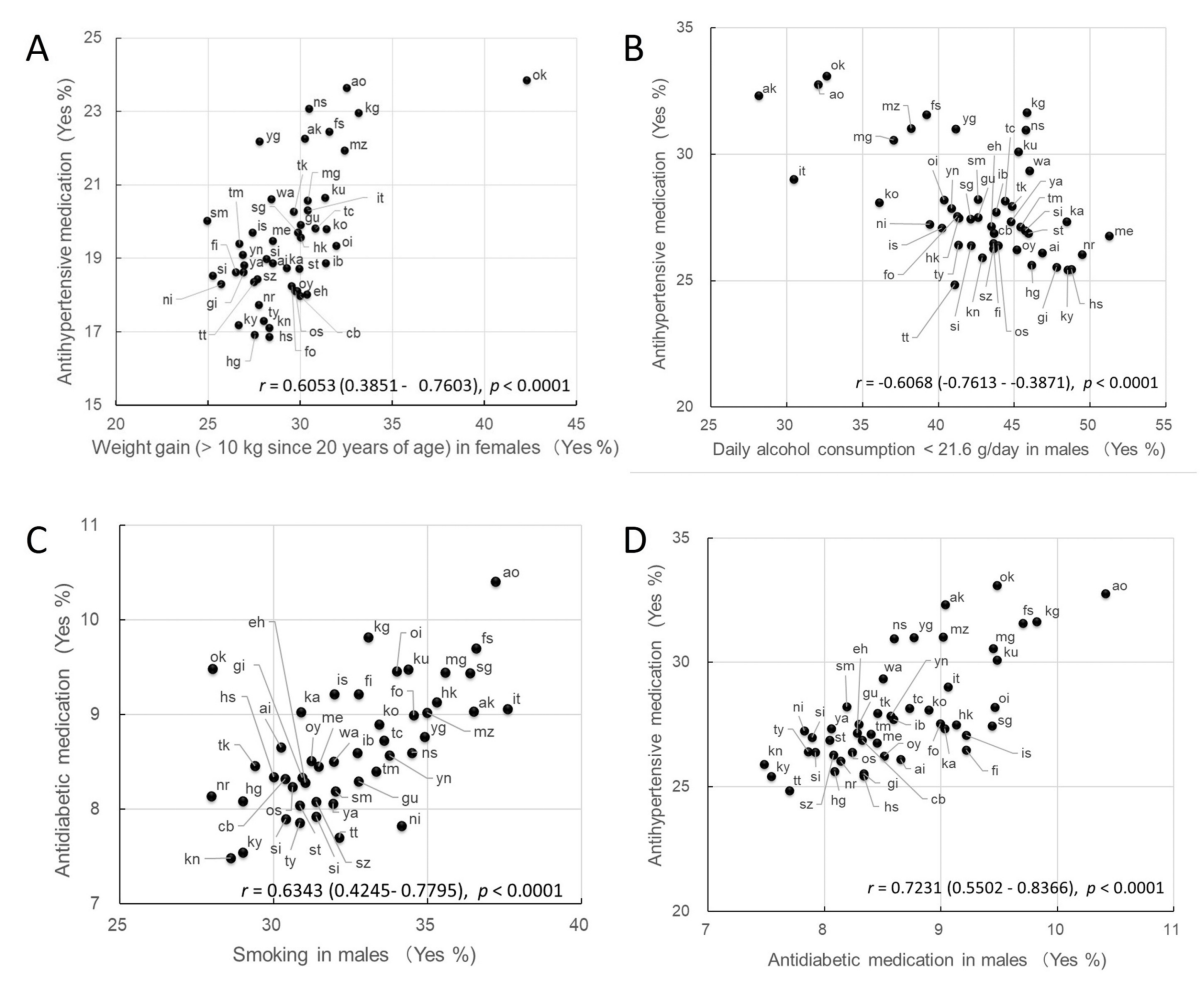
Scatter diagrams showing correlations between the variables for the prevalence of lifestyle-related disorders and lifestyle habits by prefectural units (A) Antihypertensive medication and weight gain (> 10 kg since 20 years of age) in females; (B) Antihypertensive medication and daily alcohol consumption < 21.6 g/day in males; (C) Antidiabetic medication and smoking in males; (D) Antihypertensive medication and antidiabetic medication in males ai: Aichi; ak: Akita; ao: Aomori; cb: Chiba; eh: Ehime; fi: Fukui; fo: Fukuoka; fs: Fukushima; gi: Gifu; gu: Gunma; hg: Hyogo; hk: Hokkaido; hs: Hiroshima; ib: Ibaraki; is: Ishikawa; it: Iwate; ka: Kagawa; kg: Kagoshima; kn: Kanagawa; ko: Kochi; ku: Kumamoto; ky: Kyoto; me: Mie; mg: Miyagi; mz: Miyazaki; na: Nagano; ni: Niigata; nr: Nara; ns: Nagasaki; ok: Okinawa; os: Osaka; oy: Okayama; sg: Saga; si: Shiga; sm: Shimane; st: Saitama; sz: Shizuoka; tc: Tochigi; tk: Tokushima; tm: Toyama; tt: Tottori; ty: Tokyo; wa: Wakayama; ya: Yamaguchi; yg: Yamagata; yn: Yamanashi

The correlations between several pairs of variables are presented in Figure 1.

## Discussion

This cross-sectional epidemiological study analyzed health-related open data (NDB Open Data Japan). To date, we have conducted a prefecture-based analysis of NDB Open Data Japan and showed that the prevalence of chronic pain states is tightly associated with lifestyle habits such as daily exercise levels, excessive alcohol drinking, and smoking [13], demonstrating that this research approach, that is, prefecture-based analysis of NDB Open Data Japan, works to identify lifestyle habits associated with the health issues of interest due to the lifestyle variability in each prefecture [10]. The present study focused on hypertension, diabetes, and dyslipidemia as target disorders. Lifestyle habits that correlate with the prevalence of each disorder above the set criteria are discussed below.

The prevalence of hypertension was positively correlated with the prevalence of diabetes in both males and females. This is in accordance with a previous report that demonstrated that the presence of either of the two disorders increases the risk of developing the other in individuals [16]. Another study suggested that hypertension occurs twice as frequently in patients with diabetes than in those without it [17]. One of the underlying mechanisms could be injury to blood vessels and kidneys caused by elevated blood glucose levels, resulting in high blood pressure [18]. The results of the present study also imply that common factors affect the prevalence of both hypertension and diabetes.

Slowed eating speed had a negative correlation to hypertension prevalence in females, although “similar to others” (average eating speed) was positively correlated. This result is consistent with that of a recent study demonstrating that faster eating speed was a risk factor for hypertension [19]. It has also been reported that avoiding rapid consumption is beneficial in the prevention of diabetes [20]. As hypertension prevalence was correlated with diabetes prevalence, as shown in the present study, a slow eating speed could reduce the risk of developing hypertension and diabetes in the population.

Weight gain (> 10 kg since 20 years of age) was positively correlated with the prevalence of hypertension in females. This result is in accordance with that of a systematic review that demonstrated that excessive body weight increase was associated with an increased risk of cardiovascular diseases, including hypertension [21].

Issues with chewing showed a positive correlation to hypertension prevalence in both genders, and no issues with chewing had a negative correlation in males. Consistent with this result, chewing condition, which reflects overall oral health, is associated with quality of life [22]. For example, a meta-analysis demonstrated that individuals with tooth loss have a higher risk of hypertension development [23]. The results of the present study also imply that maintaining oral health is crucial for maintaining good health and limiting the onset of hypertension.

In males, daily alcohol consumption < 21.6 and 21.6 - 43.2 g were negatively and positively correlated to the prevalence of hypertension, respectively, suggesting that the correlation between alcohol and the prevalence of hypertension is dependent on the amount. Consistent with this result, a recent meta-analysis demonstrated that alcohol consumption above 12 g/day increases the risk of hypertension. It also showed a dose-dependent effect in males, which was unclear in females, suggesting sex as a modifier of the association [24]. 

Smoking was positively correlated with the prevalence of hypertension in males. Although smoking is medically recognized as a risk factor for cardiovascular diseases, the relationship between smoking and the risk of hypertension remains controversial [25]. For example, one study demonstrated that smokers exhibited similar or slightly lower blood pressure than non-smokers, possibly due to leaner body habitus observed in smokers [26]. Another population-based study demonstrated a positive association between smoking and hypertension [27]. The results of the present study imply that smoking is a risk factor for the development of hypertension.

Daily snacking habits (every day) were negatively correlated with the prevalence of hypertension in males, implying that snacking prevents hypertension. Although unhealthy snacks high in salt, sugar, and energy and low in nutrients have a negative impact on health, including hypertension and diabetes, the influence of snacking on health seems to be dependent on snacking patterns [28]. For example, healthy snacking has been suggested to provide health benefits, including body weight regulation in adults [29]. Regarding their influence on blood pressure, a meta-analysis of prospective cohort studies demonstrated that sugar-sweetened beverages increased the risk of developing hypertension, whereas fruit and yogurt reduced the risk of hypertension [30]. Another study investigating the association between eating patterns and blood pressure showed that snacking frequency was inversely associated with hypertension prevalence [31]. These studies suggest that the risk of developing hypertension is dependent on the content and time patterns of snacking. Further studies analyzing specific snacking times and contents are necessary to clarify the effect of snacking on the prevalence of hypertension.

The prevalence of diabetes was positively correlated with smoking in males, which is consistent with the findings of a previous epidemiological study that demonstrated that smoking was an independent risk factor for the development of type 2 diabetes in males [32]. Specifically, men who smoked more than two packs of cigarettes had a 45% higher risk of developing diabetes than men who never smoked [33]. However, in females in the present study, the prevalence of diabetes was not significantly correlated with smoking. This result is consistent with that of a previous study that demonstrated an association between smoking and the risk of diabetes in both men and women; however, the effect was significantly lower in women [34]. Taken together with our study, these findings imply that the influence of smoking on diabetes differs based on sex.

Weight gain (> 10 kg since 20 years of age) was positively correlated with the prevalence of diabetes in females, which is in accordance with the perspective that obesity or excessive weight gain is a significant risk factor for poor glycemic control and the development of type 2 diabetes [35,36]. Regarding the mechanisms involved, the pathophysiology of obesity, such as increased hepatic glucose production and adipose tissue dysfunction, are suggested to be associated with the development of diabetes [37]. 　

Issues with chewing had a positive correlation with diabetes prevalence, whereas no chewing issues had a negative correlation in both genders. This is consistent with the perspective that oral health is significantly associated with the prevalence of diabetes [38]. Additionally, a cohort study identified an inverse dose-dependent association between masticatory performance and diabetes prevalence [39]. More recently, a cross-sectional study suggested that a low number of teeth and poor masticatory function increase the prevalence of type-2 diabetes [40].

Although lifestyle changes such as modifications to nutrition and exercise, smoking cessation, and limited alcohol intake have been recommended for the prevention and treatment of dyslipidemia [41], we did not observe a correlation between dyslipidemia prevalence and any of the above criteria for any single lifestyle variable in the present study. For example, weight gain was not correlated with the prevalence of dyslipidemia, although it correlated with the prevalence of hypertension and diabetes. This result is consistent with that of a recent study demonstrating that weight gain (obesity) was an independent risk factor for hypertension, diabetes, and dyslipidemia, with the effect on dyslipidemia being smaller than those for hypertension and diabetes [42]. An explanation could be that dyslipidemia develops as a result of a complex mix of factors, rather than a single risk factor. A recent cohort study demonstrated that multiple factors were associated with the development of dyslipidemia; body mass index, elevated uric acid levels, age, sleep disorders, and anxiety carried the greatest risk [43]. Additionally, in the present study, the prevalence of dyslipidemia was not significantly correlated with the prevalence of hypertension or diabetes in the population within each prefecture. This could suggest that any specific lifestyle habit associated with the prevalence of either hypertension or diabetes does not significantly influence the occurrence of dyslipidemia in a population.　

While no single lifestyle habit met the set criterion for dyslipidemia prevalence in the present study, several lifestyle habits, such as exercise in females (r = -0.3637, p = 0.0120) and alcohol drinking frequency sometimes (r = 0.4486, p = 0.0016), showed weak correlations (i.e., 0.3 < |r| ≤ 0.5) to the prevalence of dyslipidemia with statistical significance (i.e., p < 0.05). They have the possibility to be associated with dyslipidemia prevalence and warrant further investigation.

Despite its significant findings, the present cross-sectional epidemiological study using summary population data has limitations. First, the correlation analyses with a cross-sectional design only demonstrated a covariation between the variables (disorder prevalence and lifestyle habits). It should be noted that the covariation does not necessarily mean a cause-and-effect relationship between the variables. Second, the unit of data analysis was not individual but population (residents in the prefecture). The correlations between variables found in this study cannot be overgeneralized to the individual level. Third, because this study was based on self-reported data (responses to questionnaires), reporting bias may occur and influence the results. Fourth, although this study focused on the lifestyles with medium or more correlation (i.e., 0.5 < |r|), it should be noted that other lifestyles, especially those with statistical significance (i.e., p < 0.05) but low correlation (i.e., 0.3 < |r| ≤ 0.5), still have the possibility to be associated with the prevalence of hypertension, diabetes, or dyslipidemia. Fifth, the number of people eligible for specific health examinations (all individuals aged 40-74 years) in the fiscal year 2020 was approximately 54.2 million, and the number of health checkup recipients was approximately 28.9 million, resulting in a health checkup implementation rate of 53.4% [44]. Thus, there is a possibility that confounding factors exist disproportionately between health checkup recipients and non-recipients. Sixth, one of the potential confounding factors for the study utilizing the NDB Open Data Japan could be year-to-year fluctuations of data (e.g., fluctuations based on weather changes) because the data are collected and summarized annually. The analysis of datasets other than the fiscal year 2020 will be considered in the future to verify whether there are differences in correlations between years.

## Conclusions

Due to the variability of lifestyle habits of residents in each of the 47 Japanese prefectures, the prefecture-based dataset in NDB Open Data Japan is pragmatic and useful for epidemiologically investigating associations between lifestyle habits and prevalence of disorders of interest. In this study, smoking, weight gain, chewing function, eating speed, snacking, and alcohol consumption correlated with hypertension prevalence above the set criterion. Smoking, weight gain, and chewing function were correlated with the prevalence of diabetes. While no single lifestyle habit met the set criterion for dyslipidemia prevalence, several lifestyle habits showing weak correlations to dyslipidemia prevalence warrant further investigation. It would be important to raise the alarm about the lifestyle habits identified in the present study to reduce the risk of developing the corresponding disorders.
